# Titin is a new factor regulating arterial stiffness through vascular smooth muscle cell tone in male rats

**DOI:** 10.14814/phy2.70270

**Published:** 2025-03-22

**Authors:** Chaoqun Zhu, Terrance Bishop, Zachery R. Gregorich, Wei Guo

**Affiliations:** ^1^ Department of Animal Sciences University of Wyoming Laramie Wyoming USA; ^2^ Colorado State University Fort Collins Colorado USA; ^3^ Department of Animal and Dairy Sciences University of Wisconsin‐Madison Madison Wisconsin USA; ^4^ Cardiovascular Research Center University of Wisconsin‐Madison Madison Wisconsin USA

**Keywords:** angiotensin II, arterial stiffness, RNA binding protein motif 20, titin, vascular smooth muscle cell stiffness

## Abstract

Arterial stiffness is a robust predictor of cardiovascular disease and mortality. As such, there is substantial interest in uncovering its causal factors for the development of targeted treatments to regulate arterial stiffness. The elastic protein titin is a key determinant of myocardial stiffness, yet whether it plays a role in regulating arterial stiffness is unknown. In this study, we aimed to investigate the role of titin in vascular smooth muscle cell (VSMC) and overall arterial stiffness. To do this, we took advantage of rats lacking RNA binding motif 20 (RBM20), the primary splicing regulator of titin, in striated muscles. Using this model, we demonstrate that RBM20 regulates titin isoform expression in smooth muscle, with loss of the protein leading to the expression of larger titin isoforms. We show that the expression of larger titin reduces the stiffness of VSMCs. While decreased titin‐based VSMC stiffness did not affect baseline arterial stiffness, we found that arterial stiffness was reduced in response to a challenge with the potent vasoconstrictor angiotensin II (Ang II). The observed reduction in arterial stiffness following Ang II treatment was not the result of changes in either the extracellular matrix or myofilaments. We further show that the expression of a larger titin isoform ameliorates cardiac remodeling caused by Ang II‐associated hypertension. In summary, our study provides the first evidence that titin regulates VSMC stiffness, which is relevant for arterial stiffness in the context of elevated blood pressure. Furthermore, our data provide proof‐of‐concept evidence that targeting RBM20 to reduce arterial stiffness through titin isoform switching may benefit aging‐ or hypertension‐associated arterial stiffness and vascular diseases.

## 1 INTRODUCTION

The distensibility of the arterial walls is an important determinant of pulse pressure (Laurent et al., 2005; Laurent & Boutouyrie, 2020; Safar et al., 1987; Ungvari et al., 2018). Stiffening of the walls of certain arteries occurs naturally during aging (Benetos et al., 1993; Mitchell et al., 2004; van der Heijden‐Spek et al., 2000) and is associated with cardiovascular diseases (CVDs), such as hypertension and aortic aneurysms (Laurent et al., 2006; Sehgel et al., 2013; Sehgel, Sun, et al., 2015; Sehgel, Vatner, & Meininger, 2015). As such, arterial stiffness has been proposed as a new therapeutic target for the treatment of CVDs (Agabiti‐Rosei et al., 2009; Boutouyrie et al., 2011; Schiffrin, 2010). Studies have shown that the endothelium (Safar et al., 2001; Wallace et al., 2007), extracellular matrix (ECM) (Dao et al., 2005; Fleenor et al., 2010, 2012; Greenwald, 2007; Humphrey et al., 2014; Lacolley, Li, et al., 2017; Lemarié et al., 2010; Valentín et al., 2011; Wagenseil & Mecham, 2012; Zulliger & Stergiopulos, 2007), and vascular smooth muscle cells (VSMCs) (Lacolley, Regnault, et al., 2017; Qiu et al., 2010; Sehgel et al., 2013; Sehgel, Sun, et al., 2015; Sehgel, Vatner, & Meininger, 2015) play a role in determining vascular arterial stiffness. VSMCs comprise much of the arterial medial layer and are the main constituents of the vascular wall. Yet, the molecular mechanisms regulating VSMC tone remain incompletely understood.

Titin is a giant sarcomeric protein that plays a critical role in sarcomere assembly and the maintenance of the structural integrity of the myofibril in striated muscles (Clark et al., 2002; Granzier & Labeit, 2004; Labeit & Kolmerer, 1995; Tskhovrebova & Trinick, 2003). It also plays a key role in determining myocardial stiffness by virtue of its elastic properties (Granzier & Labeit, 2002, 2004; Guo et al., 2010; Hamdani & Paulus, 2013; Labeit & Kolmerer, 1995). Titin's elasticity is largely dependent on its size changes due to isoform switching (Cazorla et al., 2000; Granzier & Labeit, 2002; Guo et al., 2010). Titin is encoded by a single gene containing 363 coding exons (Bang et al., 2001). In the heart, alternative splicing produces two classes of titin isoforms: the larger and more compliant N2BA isoforms and a small and stiff N2B isoform (Bang et al., 2001; Cazorla et al., 2000; Granzier & Labeit, 2002, 2004; Greaser et al., 2008; Guo et al., 2010; Guo & Sun, 2018; Hamdani & Paulus, 2013; Labeit & Kolmerer, 1995; Stroik et al., 2024). Numerous studies in human patients and animal models have shown that switching between N2BA and N2B isoforms contributes to ventricular wall stiffness, with an elevated ratio of N2BA to N2B titin decreasing myocardial stiffness (Bell et al., 2000; Borbély et al., 2009; Hamdani et al., 2013; Hudson et al., 2011; Makarenko et al., 2004; Nagueh et al., 2004; Neagoe et al., 2002; Shapiro et al., 2007; Warren, Jordan, et al., 2003; Wu et al., 2002). In addition to titin expression in striated muscles, studies have shown that 700 kDa and 2000 kDa titin‐like proteins are expressed in chicken vascular and visceral smooth muscles, as well as in adult gizzard smooth muscle (Chi et al., 2005; Kim & Keller, 2002; Maher et al., 1985; Turnacioglu et al., 1997). Later studies using a titin exon array and Western blot employing titin antibodies in human and pig smooth muscle identified two titin isoforms (Labeit et al., 2006). One isoform is approximately 1000 kDa, and the other is similar in size to titin expressed in human soleus muscle (~3700 kDa) (Labeit et al., 2006). However, it is completely unknown whether titin plays a role in determining VSMC stiffness and thus contributes to overall arterial stiffness.

Our early work identified a splicing factor, known as RNA binding motif 20 (RBM20), that regulates titin splicing in a dosage‐dependent manner (Guo et al., 2012). RBM20 mediates the exclusion of exons primarily in titin's I‐band region such that the production of short N2B titin is favored in the heart. If RBM20 expression is either reduced or lost, the expression of larger N2BA titin isoforms is favored, with a giant non‐physiological N2BA titin isoform expressed as the sole isoform in *Rbm20* knockout (KO) rats (Guo et al., 2012). When larger N2BA isoforms are increased in the *Rbm20* KO animals, the cardiac muscle becomes more compliant (Greaser et al., 2008; Guo et al., 2013, 2021; Methawasin et al., 2016). Taking advantage of this model, herein we sought to determine if RBM20 modulates titin isoform switching in smooth muscle; if titin‐based changes in VSMC tone impact overall arterial stiffness; and whether modulation of arterial stiffness through the RBM20‐titin axis is beneficial for hypertension‐induced cardiac hypertrophy and remodeling. To accomplish the latter, we employed treatment with angiotensin II (Ang II), a hormone that increases blood pressure through vasoconstriction, to induce hemodynamic overload and stimulate cardiac hypertrophy.

## 2 MATERIALS AND METHODS

### 2.1 Experimental animals and Ang II treatment

*Rbm20* KO rats were cross‐bred from Sprague–Dawley (SD) × Brown Norway (BN). All experimental animals (WT and KO) were maintained on Envigo Teklad rodent diet (cat# 8604; Indianapolis, IN). Male WT and KO rats (10 per group), age 6–7 weeks, were randomly subjected to normal saline infusion (control) or Ang II (cat# A9525; Sigma‐Aldrich, St. Louis, MO) infusion at a dose of 400 ng/kg/min by subcutaneously implanting osmotic minipumps (Osmotic pump model 2004, Alzet®, Cupertino, CA) for 28 days. This study was carried out in strict accordance with the recommendations in the Guide for the Care and Use of Laboratory Animals from the National Institutes of Health. All procedures were approved by the Institutional Animal Use and Care Committee of the University of Wyoming.

### 2.2 Blood pressure measurement, and echocardiography

Blood pressure was measured by tail cuff system (Kent Scientific, Torrington, CT) in conscious rats 1 day before the start of minipump implantation (day 0, considered as baseline), and then at days 7, 14, 21, and 28 of the 4‐week experimental period. Cardiac geometry and function were evaluated under anesthesia using 2‐dimensional guided M‐mode echocardiography (Philips SONOS 5500, Phillips Medical Systems, Chicago, IL) equipped with a 15 to 6 MHz linear transducer (Phillips Medical Systems) at days 0 and 28. The detailed procedure for echocardiographic assessment is described in our previous publication (Guo et al., 2021).

### 2.3 Pulse wave velocity (PWV) measurement

Male WT and KO rats (10 per group), age 6–7 weeks, were infused with normal saline or Ang II for 28 days. Aortic PWV was measured by Doppler system (MouseDoppler data acquisition system, Indus Instruments, Webster, TX) 1 day before the start of minipump implantation (day 0, considered as baseline), and then on days 7, 14, 21, and 28 of the 4‐week infusion period. Rats were anesthetized with a xylazine and ketamine cocktail and positioned supine on a heating board with limbs secured to ECG electrodes. Pulse waves were detected at the transverse aortic arch and the abdominal aorta using Doppler probes (20 Hz). The time elapsed between the ECG R‐wave and the foot of the Doppler signal was determined for each site, and PWV was calculated using the following equation:

$$\mathrm{PWV}=\left(\text{distance between probes}\right)/\left(\Delta_{\text{time abdominal}}-\Delta_{\text{time transverse}}\right).$$

### 2.4 Biochemical assays

Collagen content was quantified by measuring total hydroxyproline content in heart tissues. In brief, the heart samples were hydrolyzed with 6 N HCl at 100°C for 16 h. The hydrolyzed samples were evaporated using a Speedvac concentrator and rehydrated in Milli‐Q water. Next, chloramine T solution was added and incubated for 20 min. Subsequently, 3.15 M perchloric acid was added and incubated for 5 min. p‐DMAB (cat# D2004; Sigma‐Aldrich) solution was added and incubated at 60°C for 20 min. Finally, the absorbance was recorded at 557 nm using a spectrophotometer. The total tissue hydroxyproline content is reported in μg per mg wet tissue.

### 2.5 Immunohistochemistry and histology

Aorta and heart tissues were collected for cryosection preparation. 7 μm thick aorta sections were prepared and blocked using 10% normal goat serum (cat# 50062Z; Thermo Fisher Scientific, Waltham, MA) and then incubated with primary antibodies against titin (anti‐titin M‐line, home‐made (Sebestyén et al., 1995; Trombitás et al., 1998; Trombitás et al., 1999; Wang & Greaser, 1985)) or α‐smooth muscle actin (cat# A2547; Sigma‐Aldrich) diluted in 10% normal goat serum (cat# 50062Z; Thermo Fisher Scientific) overnight at 4°C. Primary VSMCs were cultured on cover slides and fixed with −20°C methanol for 5 min. VSMCs were blocked in 10% normal goat serum (cat# 50062Z; Thermo Fisher Scientific) and then incubated with primary antibodies against titin (anti‐titin PEVK, M‐line, Z‐line and C‐zone, home‐made (Greaser et al., 2000; Sebestyén et al., 1995; Trombitás et al., 1998; Trombitás et al., 1999; Wang & Greaser, 1985)) or α‐smooth muscle actin (cat# A2547; Sigma‐Aldrich) diluted in 10% normal goat serum (cat# 50062Z; Thermo Fisher Scientific) overnight at 4°C. Membranes were subsequently stained with goat anti‐Mouse IgG (H + L), Alexa Fluor™ 488 (cat# A‐11001, Thermo Fisher Scientific) and goat anti‐rabbit IgG (H + L), Alexa Fluor™ 594 (cat# A‐11012, Thermo Fisher Scientific). DAPI was used to stain the nucleus.

Tissues were fixed in 4% paraformaldehyde buffered with phosphate‐buffered saline (PBS), routinely dehydrated, and embedded in paraffin. 5 μm thick sections were cut and stained with Masson's trichrome as previously described (Guo et al., 2012). Wheat germ agglutinin (cat# W11261; Thermo Fisher Scientific) staining was performed on heart sections to measure the size of cardiomyocytes.

### 2.6 Primary VSMC cultures and stiffness measurement through atomic force microscopy (AFM)

Primary VSMCs were isolated from the thoracic aorta of WT and KO rats (150–200 g, 6‐week‐old) using enzymatic digestion (Sreejayan & Yang, 2007). After isolation, VSMCs were plated onto glass‐bottomed cell culture dishes (WillCo Wells B.V., Amsterdam, Netherlands), and VSMC stiffness was determined by a Bruker Resolve AFM coupled to a confocal microscope, using a nanoindentation protocol. Individual VSMCs were probed through an indentation depth over a range of 100–300 nm using a probe tip (Mode: PFQNM‐LC‐A‐CAL; Bruker, Billerica, MA). Force curves were continuously collected for a 2 min duration for each cell. The force curves were analyzed using Bruker's NanoScope Analysis software. The mean of elastic stiffness values was calculated from Young's elastic modulus (E) of each cell and then averaged together for each group.

### 2.7 RBM20 overexpression and VSMC stiffness measurement

Primary *Rbm20* KO VSMCs isolated and cultured as described above were infected with adenovirus carrying RBM20 (Ad‐RBM20). VSMCs infected with adenovirus carrying GFP (Ad‐GFP) were used as the control group. Adenoviruses were produced by General Biosystems, Inc. (Durham, NC). Forty‐eight hours after infection, VSMCs were individually probed using AFM, as described above. Each time, KO VSMCs isolated from two animals were mixed and separated into 2 dishes for Ad‐RBM20 and Ad‐GFP treatment, respectively. A total of six animals were used.

### 2.8 Protein extraction and Western blot analysis

Heart and aorta samples were homogenized in urea‐thiourea lysis buffer (8 M urea, 2 M thiourea, 75 mM DTT, 3% SDS, 0.05% bromophenol blue, 0.05 M Tris–HCl, pH 6.8) as described previously (Warren, Krzesinski, & Greaser, 2003; Zhu & Guo, 2017). Protein extracts were separated by SDS‐PAGE gel and transferred onto PVDF membranes. Membranes were then probed with antibodies against titin (anti‐titin PEVK region 9D10, home‐made (Greaser et al., 2000)), RBM20 (home‐made (Guo et al., 2012)), myosin light chain (cat# 3672; Cell Signaling Technology, Danvers, MA), phosphorylated myosin light chain (cat# 3671; Cell Signaling Technology), or α‐smooth muscle actin (cat# A2547; Sigma‐Aldrich). Histone 3 (cat# 9715; Cell Signaling Technology) and GAPDH (cat# 2118; Cell Signaling Technology) served as nuclear and cytoplasmic protein loading controls, respectively. Goat anti‐rabbit‐HRP (cat# W401B; Promega, Madison, WI) and goat anti‐mouse‐HRP (cat# 7076; Cell Signaling Technology) were used as secondary antibodies.

### 2.9 Titin isoform detection using gel electrophoresis

Titin isoforms were resolved using a vertical SDS‐1% agarose gel electrophoresis (VAGE) system (Warren, Krzesinski, & Greaser, 2003; Zhu & Guo, 2017). Protein bands were visualized by silver staining, or protein bands were cut and transferred to PVDF membranes for immunoblotting using an antibody against titin (home‐made (Trombitás et al., 1999; Wang & Greaser, 1985)).

### 2.10 RNA extraction and RT‐PCR analysis

Aorta tissues were pulverized in liquid nitrogen, and then total RNA was extracted using TRIzol Reagent (cat# 15596026; Thermo Fisher Scientific) according to the manufacturer's method. After extraction, RNA was treated with DNase I (cat# AMPD1; Sigma‐Aldrich) to remove genomic DNA contamination. 1 μg of total RNA was reverse transcribed with the ImProm‐II™ Reverse Transcriptase System (cat# A3802; Promega) using random primers. The reaction was used as a template for PCR using Taq DNA polymerase (cat# F93481‐1; Lucigen, Middleton, WI) and primers spanning spliced exons (Table S1) to examine mRNA splicing.

### 2.11 Statistical analysis

GraphPad prism software was used for statistical analysis. Results are presented as mean ± SEM. Statistical significance between two groups was determined using an unpaired Student's *t* test. *p* < 0.05 was considered statistically significant.

## 3 RESULTS

### 3.1 RBM20 regulates titin splicing in VSMCs

**FIGURE 1:**
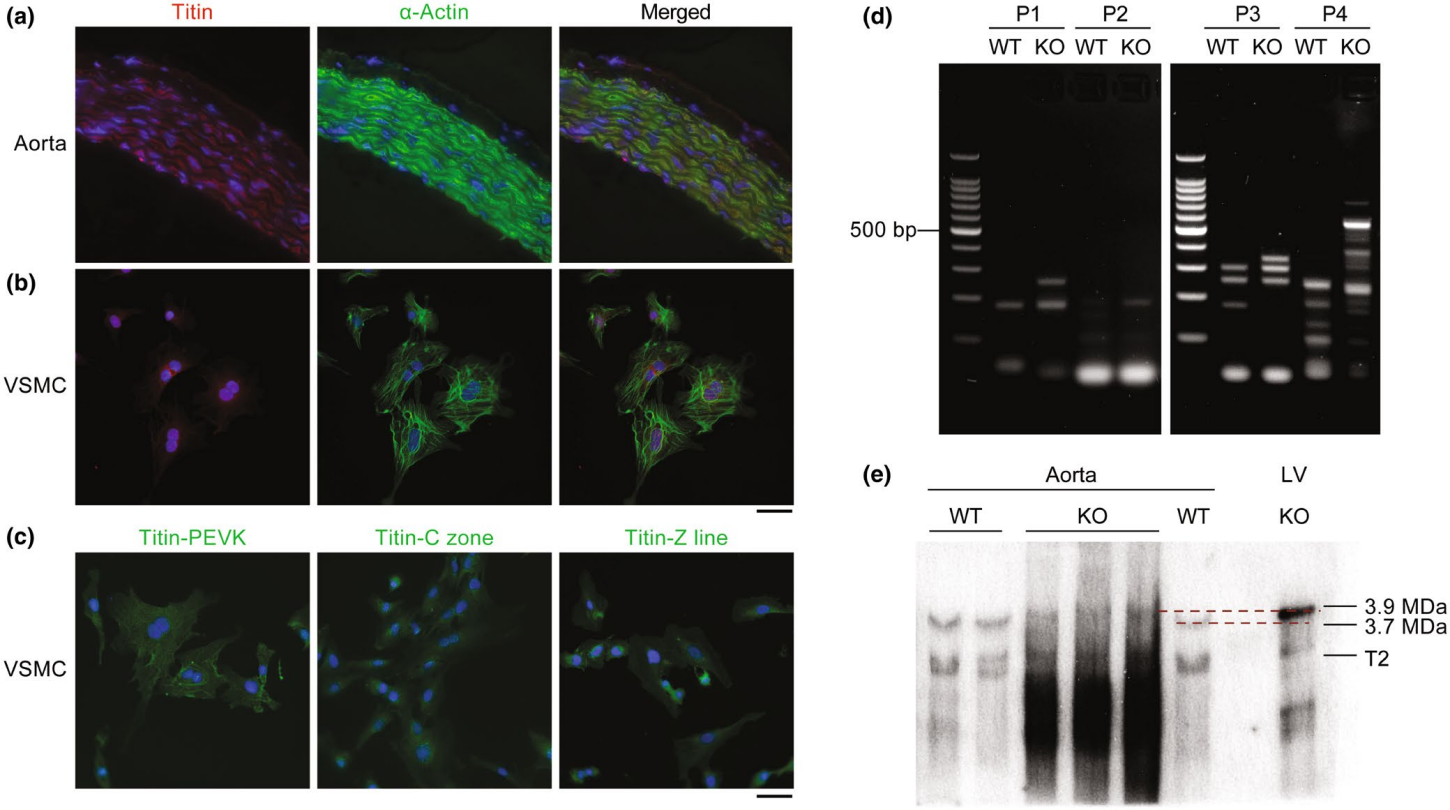
Titin expression in aortic smooth muscle cells. (a) Titin immunostaining in aorta from wildtype (WT) rats. Titin (red), α‐Actin (green), and DAPI (blue). (b) Titin immunostaining in vascular smooth muscle cells (VSMCs) isolated from WT rats. Titin (red), α‐Actin (green), and DAPI (blue). (c) Titin immunostaining in isolated VSMCs. Titin (green) and DAPI (blue). Scale bars: Aorta, 50 μm; VSMC, 100 μm. (d) Smooth muscle titin different mRNA splicing patterns between WT and *Rbm20* knockout (KO) detected by RT‐PCR. (e) Western blot analysis of titin isoform expression in aortic smooth muscle. The T2 band corresponds to a fragment of titin encompassing titin's A‐band section and ~ 100–200 kDa its distal I‐band section (Opitz et al., 2004).

To determine the localization of titin in smooth muscle, we performed immunostaining using an antibody against the titin M‐line in intact rat aorta tissue, as well as primary cultured rat VSMCs. In intact aorta, antibody labeling was clearly located in the middle (tunica media), but not in the inner (tunica intima) or outer (tunica adventitia) layers (Figure 1A). In isolated VSMCs, immunostaining with the M‐line titin antibody showed that titin is in the cytoplasm of the cells (Figure 1B). Moreover, antibodies against the titin PEVK region, Z‐line, and C‐zone also stained titin in the cytoplasm of VSMCs (Figure 1C).

In striated muscle, RBM20 regulates titin size by promoting the exclusion of exons in titin's I‐band region such that production of shorter and less compliant titin isoform is favored (Greaser et al., 2000; Guo et al., 2012; Li et al., 2012, 2013; Maimaiti et al., 2021). However, it is unknown if and/or to what extent RBM20 regulates titin splicing in smooth muscle. To investigate this, we performed RT‐PCR with primers designed to amplify RBM20‐regulated exons (120–122, 146–148, 148–150, and 194–196) in titin's I‐band region in aortic smooth muscle isolated from WT and *Rbm20* KO rats (Greaser et al., 2000). All of these primers amplified larger bands in *Rbm20* KO heart muscle compared to WT heart muscle (Li et al., 2012, 2013). These results indicated that a greater number of exons in the I‐band region of titin are included when RBM20 is absent from smooth muscle (Figure 1D). Some PCR products show multiple larger bands which are consistent with the splicing pattern identified in striated muscles due to multiple splicing events, including exon shuffling and intron retention (Li et al., 2012, 2013). The results indicate that more titin exons are included in *Rbm20* KO smooth muscle.

To confirm that the inclusion of additional exons in titin's I‐band increases titin size in smooth muscle, we compared titin size in aortic smooth muscles from WT and *Rbm20* KO rats using 1% SDS‐agarose gel electrophoresis (Warren, Krzesinski, & Greaser, 2003; Zhu & Guo, 2017). Left ventricular (LV) myocardium from *Rbm20* KO rats was employed as a reference for protein size estimation because, in *Rbm20* KO LV, a single large titin isoform with a molecular weight of ~3.9 MDa is expressed (Guo et al., 2012). Western blot analysis using an anti‐titin antibody (Greaser et al., 2000) that reacts with the PEVK region revealed that titin size increased from ~3.7 MDa in WT to ~3.9 MDa (the most abundant band on the top; similar to that in cardiac muscle) in KO rat smooth muscle (Figure 1E). The T2 band and the intense smear band lower than the T2 band are considered degraded titin (Figure 1E). These data further indicate that RBM20 regulates titin splicing in smooth muscle, with the loss of RBM20 promoting the expression of larger titin isoforms.

### 3.2 The expression of larger titin decreases VSMC stiffness

**FIGURE 2:**
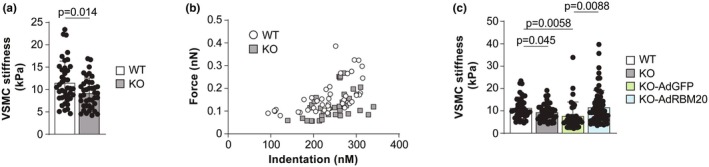
*Rbm20* KO reduces stiffness of VSMCs. (a) Computed elastic stiffness of individual VSMCs, as determined by atomic force microscopy (AFM) measurements in WT and *Rbm20* KO rats. *n* = 40 total cells from 4 rats per group. (b) Distribution of force as a function of indentation in WT and *Rbm20* KO VSMCs measured by AFM. *n* = 35–43 cells from 4 rats per group. (c) Elastic stiffness of individual VSMC after re‐expression of RBM20 in *Rbm20* KO VSMCs by adenovirus‐RBM20 infection. Adenovirus‐GFP served as a negative control. *n* = 35–68 cells per group. Bar graphs show mean ± SD. Data points represent measurements from individual cells. Significance determined by pairwise comparisons with unpaired Student's *t* test.

Given that titin plays an important role in determining the stiffness of cardiomyocytes (Cazorla et al., 2000; Granzier & Labeit, 2002, 2004; Guo et al., 2010; Hamdani & Paulus, 2013; Labeit & Kolmerer, 1995), we next sought to determine whether the expression of larger titin impacts the stiffness of VSMCs. AFM was used to assess the stiffness of individual VSMCs isolated from aortic smooth muscle in WT and *Rbm20* KO rats. In VSMCs from *Rbm20* KO rats, the elastic modulus was significantly lower than that in VSMCs from WT rats (Figure 2A,B). These results suggest that titin size plays an important role in determining the stiffness of VSMCs. To further confirm that titin plays an integral role in determining VSMC stiffness, RBM20 was expressed in VSMCs isolated from *Rbm20* KO rats through adenoviral transduction. VSMCs infected with adenovirus carrying GFP served as controls. Forty‐eight hours post‐infection, the stiffness of individual VSMCs was determined using AFM. The expression of RBM20, but not GFP, recovered the elastic modulus in *Rbm20* KO VSMCs to the WT level (Figure 2C). Collectively, these results indicate that titin size plays a crucial role in determining the stiffness of VSMCs, with the expression of larger titin decreasing VSMC stiffness.

### 3.3 Decreased titin‐based VSMC stiffness ameliorates Ang II‐induced arterial stiffness

**FIGURE 3:**
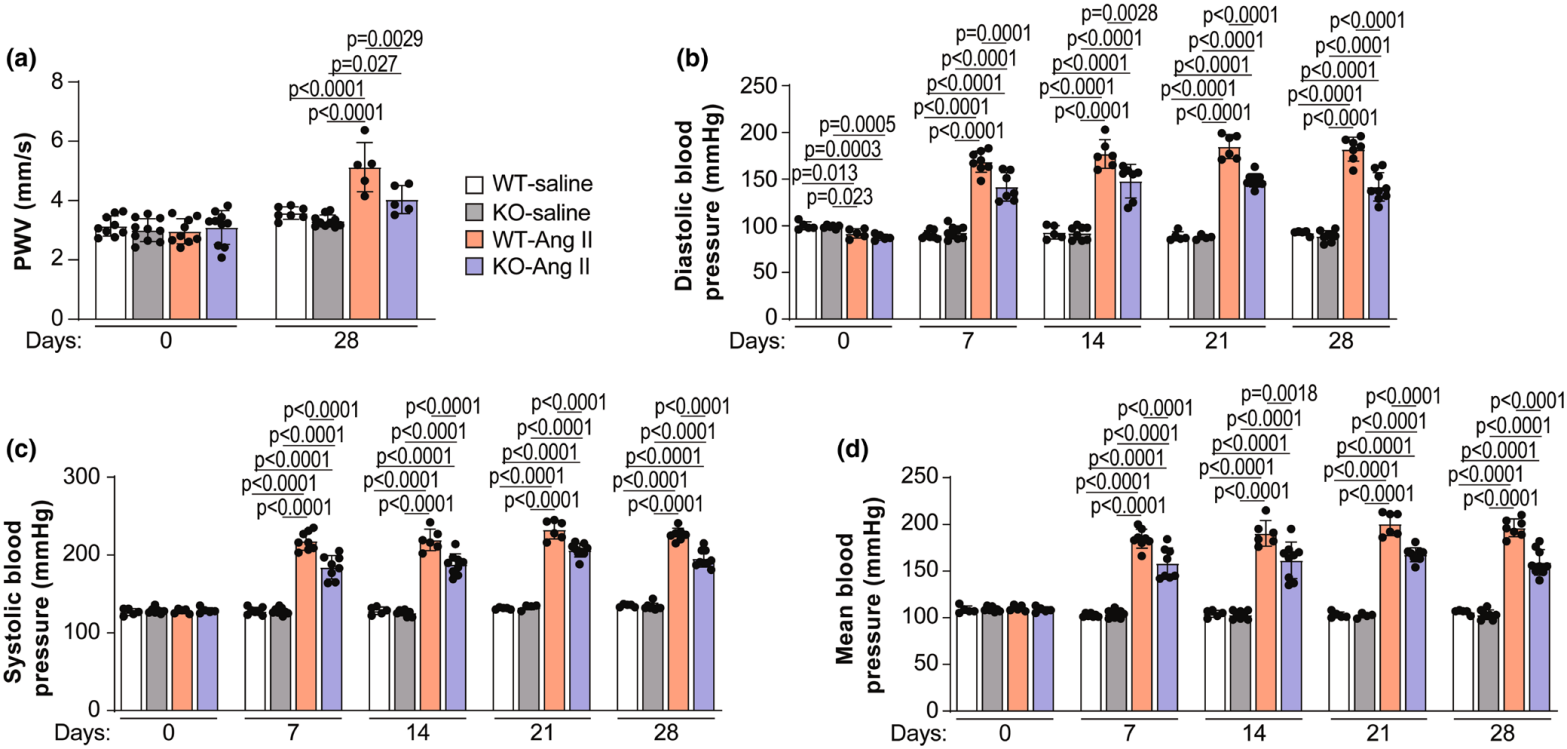
*Rbm20* KO ameliorates angiotensin II (Ang II)‐induced hypertension and aortic stiffness. (a) Pulse wave velocity (PWV) measured by Doppler System. *n* = 5–10 animals per group. Diastolic blood pressure (b), systolic blood pressure (c), and mean blood pressure (d). *n* = 4–9 animals per group. Bar graphs show mean ± SD. Data points represent measurements from individual animals. Significance determined by one‐way ANOVA followed by the Tukey post hoc test.

We next investigated whether decreased titin‐based VSMC stiffness affects overall arterial stiffness. To do this, we measured PWV, a direct measure of arterial stiffness (Mackenzie et al., 2002), in WT and *Rbm20* KO rats. At baseline, there was no difference in PWV between WT and *Rbm20* KO rats (Figure 3A). To determine whether the decrease in titin‐based VSMC stiffness is relevant in the context of hypertension, WT and *Rbm20* KO rats were challenged with continuous infusion of the vasoconstrictor Ang II (400 ng/kg/min) over a period of 28 days. In WT rats treated with Ang II, PWV was dramatically increased at day 28 compared to that in WT rats treated with saline (Figure 3A). In *Rbm20* KO rats, PWV was also elevated at day 28 compared to that in KO‐saline rats, although the increase was less than in the WT group (PWV was significantly lower in KO‐Ang II versus WT‐Ang II rats) (Figure 3A).

To further evaluate if compliant vessel walls protect blood pressure from Ang II challenge, we measured the blood pressure in conscious rats by noninvasive tail‐cuff. We observed that at the basal level, blood pressure showed no difference between WT and KO rats (Figure 3B–D). After Ang II infusion for 7 days, blood pressure was significantly increased in both WT and KO rats compared to the respective saline‐treated groups (Figure 3B–D). This was the case over the entire treatment period (28 days). Moreover, blood pressure in Ang II‐treated *Rbm20* KO rats was significantly lower than in Ang II‐treated WT rats over the 28‐day treatment period (Figure 3B–D). Collectively, these results indicate that titin‐based alterations in VSMC tone impact arterial stiffness in the context of Ang II‐associated hypertension.

### 3.4 Decreased titin‐based VSMC tone minimally impacts Ang II‐induced vascular remodeling

**FIGURE 4:**
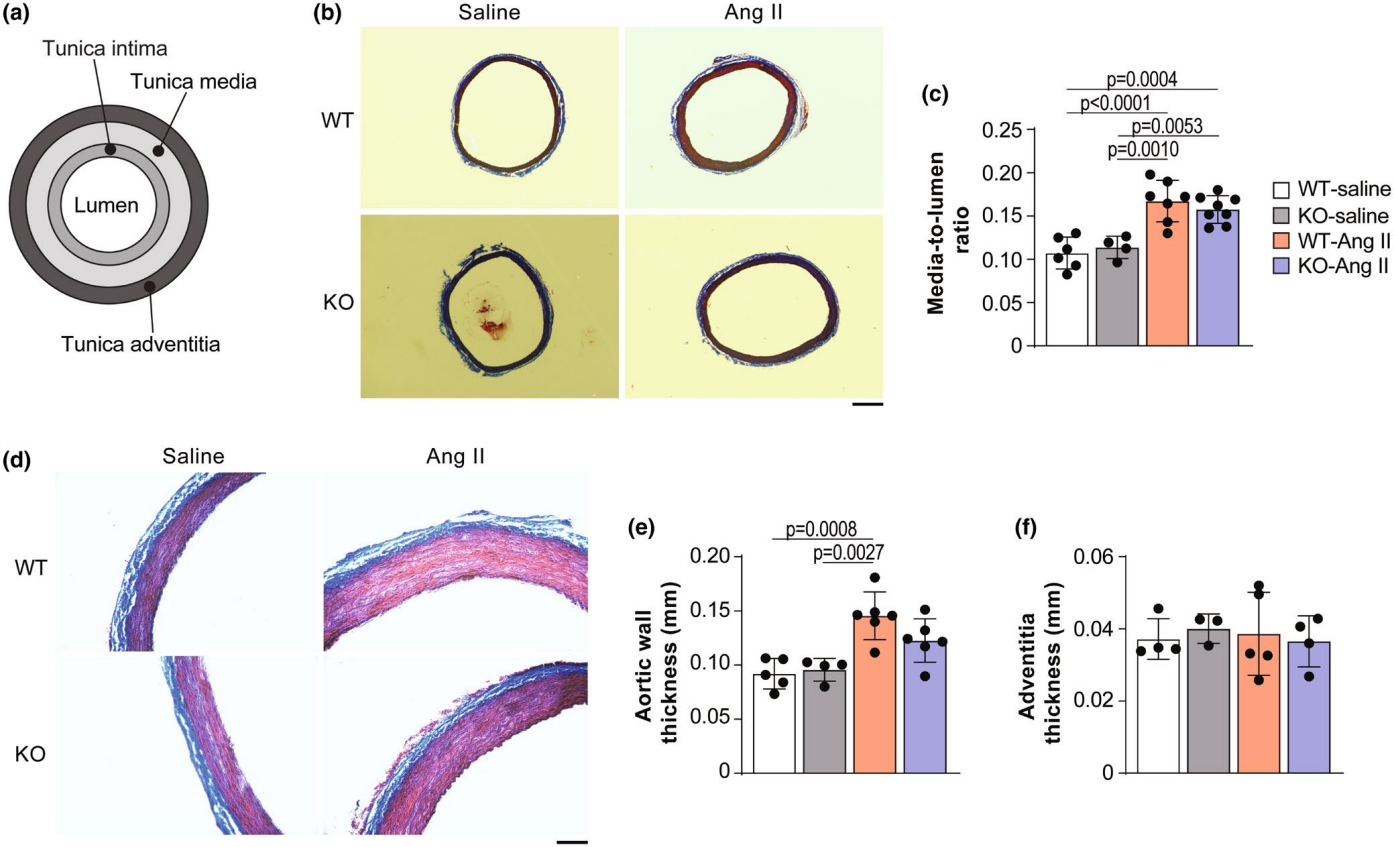
*Rbm20* KO does not affect vascular remodeling in response to Ang II treatment. (a) Schematic showing a simplified artery structure. Aorta sections (b) were stained and quantified for media‐to‐lumen ratio (c). Scale bar, 500 μm. *n* = 4–8 animals per group. Aorta sections were stained for fibrosis with trichrome staining (d) and quantified (e, f). Scale bar, 100 μm. *n* = 3–6 animals per group. Bar graphs show mean ± SD. Data points represent measurements from individual animals. Significance determined by one‐way ANOVA followed by the Tukey post hoc test.

Vascular remodeling, such as changes in the thickness of the vascular wall and changes in the ECM, is a well‐established determinant of arterial stiffness (Dao et al., 2005; Fleenor et al., 2010, 2012; Greenwald, 2007; Humphrey et al., 2014; Lemarié et al., 2010; Valentín et al., 2011; Wagenseil & Mecham, 2012; Zulliger & Stergiopulos, 2007). To confirm that changes in titin are responsible for the observed reduction in arterial stiffness following Ang II treatment in *Rbm20* KO rats, we next sought to rule out potential contributions from vascular remodeling. Quantification of the tunica media‐to‐lumen diameter ratio in Masson's trichrome‐stained aorta tissue revealed an increased ratio in WT and *Rbm20* KO rats treated with Ang II compared to the untreated group, but no difference was observed between either treated or untreated WT and KO groups, respectively (Figure 4A–C). Ang II infusion increased aortic wall thickness, but the increase was only significant in Ang II‐treated WT animals (Figure 4D,E). Interestingly, the thickness of the tunica adventitia layer (consisting predominantly of ECM) did not change between WT and *Rbm20* KO with or without Ang II treatment (Figure 4D,F). These results suggest that decreased arterial stiffness in response to Ang II treatment in *Rbm20* KO rats may be partly due to lessened thickening of the arterial walls.

### 3.5 Changes in actomyosin cross‐bridges in response to Ang II treatment are similar in WT and *Rbm20* KO smooth muscle

**FIGURE 5:**
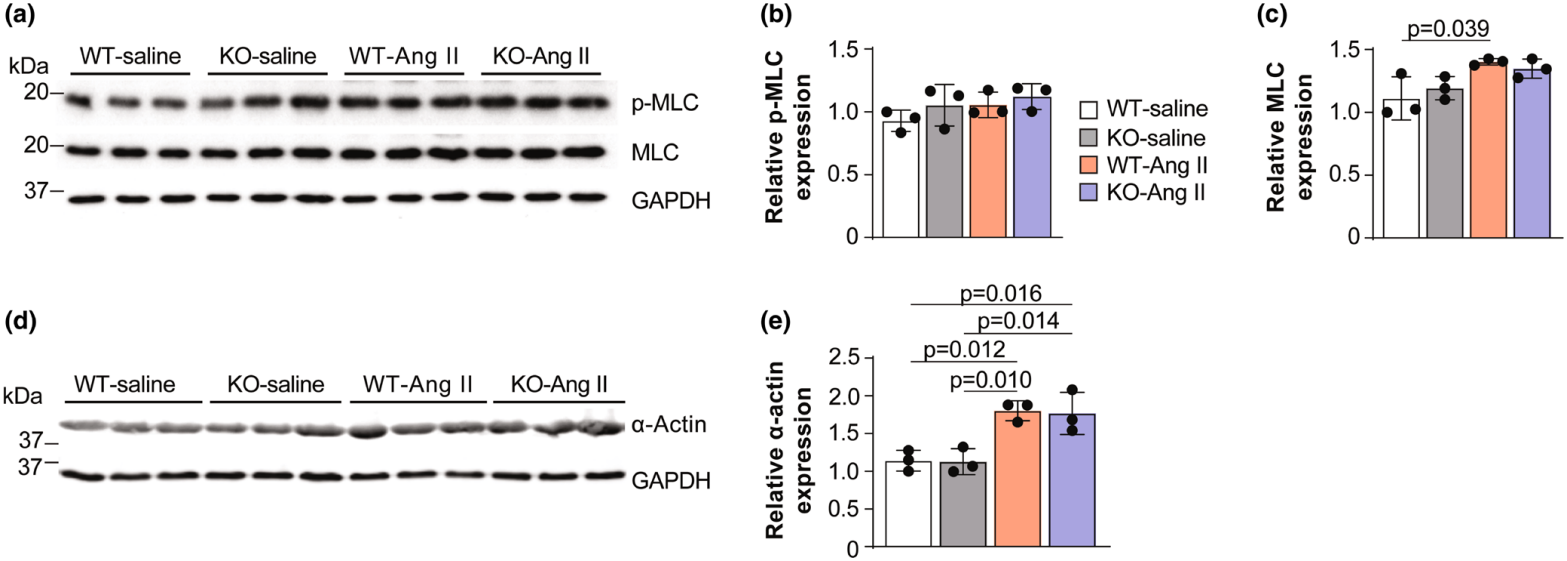
*Rbm20* Ko has little effect on the expression of contractile proteins in aorta. (a–e) Expression of phosphorylated myosin light chain (p‐MLC), myosin light chain (MLC), and α‐smooth muscle Actin in WT and KO control (saline) and Ang II‐treated groups with quantification. *n* = 3 animals per group. Bar graphs show mean ± SD. Data points represent measurements from individual animals. Significance determined by one‐way ANOVA followed by the Tukey post hoc test.

Actomyosin cross‐bridge formation is another factor that can contribute to the stiffness of VSMCs. To determine the molecular mechanisms leading to VSMC stiffness change in *Rbm20* KO rats, we investigated whether mediators of the VSMC contractile process would have effects on VSMC stiffness. The actin cytoskeleton in the VSMC is a core machinery to regulate arterial stiffness, and the phosphorylation of myosin light chain is a trigger of actomyosin cross‐bridge formation (Sebestyén et al., 1995). Therefore, we measured the expression and phosphorylation status of myosin light chain, as well as the expression of α‐smooth muscle actin, in aortas from WT and *Rbm20* KO rats using Western blot. The phosphorylation of myosin light chain was not different in WT and *Rbm20* KO aortas and did not change in response to Ang II treatment (Figure 5A,B). There was a slight, albeit statistically significant, increase in MLC expression in Ang II‐treated versus saline‐treated WT animals (Figure 5A,C). On the other hand, while the expression of α‐actin increased in both WT and *Rbm20* KO aortas following Ang II treatment, the increase was comparable (Figure 5D,E). These results further suggest that changes in titin size, not the contractile machinery, are responsible for the observed increase in arterial compliance in Ang II‐treated *Rbm20* KO rats.

### 3.6 Increased titin‐based compliance in *Rbm20* KO rats mitigates cardiac hypertrophy and fibrosis in response to Ang II‐induced hypertension

**FIGURE 6:**
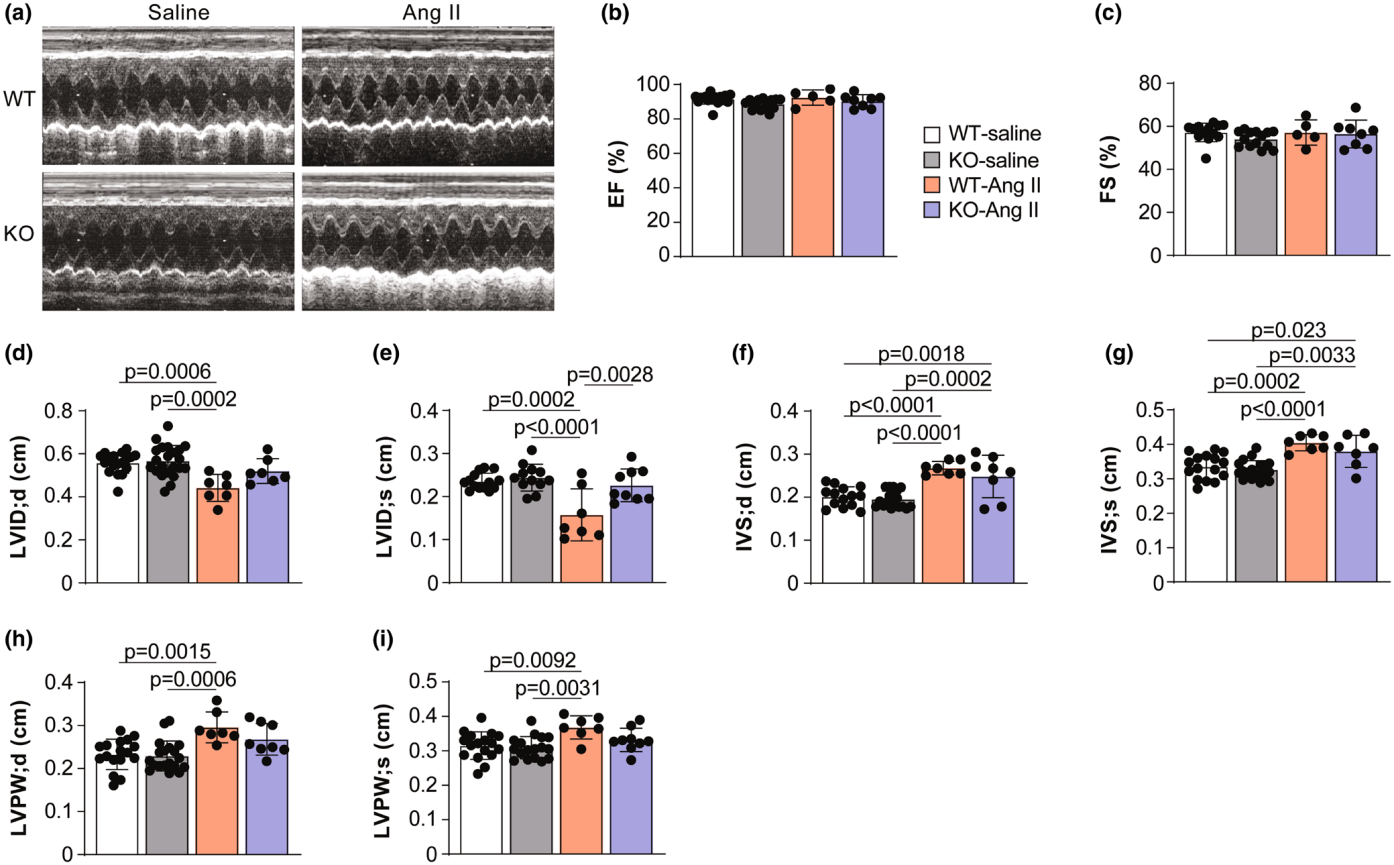
*Rbm20* KO alleviates Ang II‐induced cardiac hypertrophy. (a) Representative echocardiographic pictures. Quantification of EF (ejection fraction) (b), FS (fractional shortening) (c), LVID;d (left ventricular end diastolic internal diameter) (d), LVID;s (left ventricular end systolic internal diameter) (e), IVS;d (interventricular septum dimension in diastole) (f), IVS;s (interventricular septum dimension in systole) (g), LVPW;d (left ventricular posterior wall thickness in diastole) (h), and LVPW;s (left ventricular posterior wall thickness in systole) (i). *n* = 5–22 animals per group. Bar graphs show mean ± SD. Data points represent measurements from individual animals. Significance determined by one‐way ANOVA followed by the Tukey post hoc test.

Next, we evaluated whether increased arterial compliance in Ang II‐treated *Rbm20* KO animals benefits hypertension‐induced cardiac remodeling. Cardiac function was assessed by echocardiography (Figure 6A). Systolic function was similar in WT and *Rbm20* KO rats and did not change following Ang II treatment, as evidenced by no difference in either ejection fraction (EF) or fractional shortening (FS) between the groups (Figure 6B,C). Consistent with the development of hypertrophy in Ang II‐treated WT rats, the internal diameter of the left ventricle decreased at end diastole (LVID;d) and systole (LVID;s) (Figure 6D,E). In Ang II‐treated *Rbm20* KO rats, this decrease was blunted, indicating a reduction in Ang II‐induced cardiac hypertrophy in these animals (Figure 6D,E). The Ang II‐associated increase in septal thickness was comparable between WT and Rbm20 KO rats (Figure 6F,G). The thickness of the posterior wall of the LV was also significantly elevated in WT but not *Rbm20* KO rats following Ang II treatment (Figure 6H,I).

**FIGURE 7:**
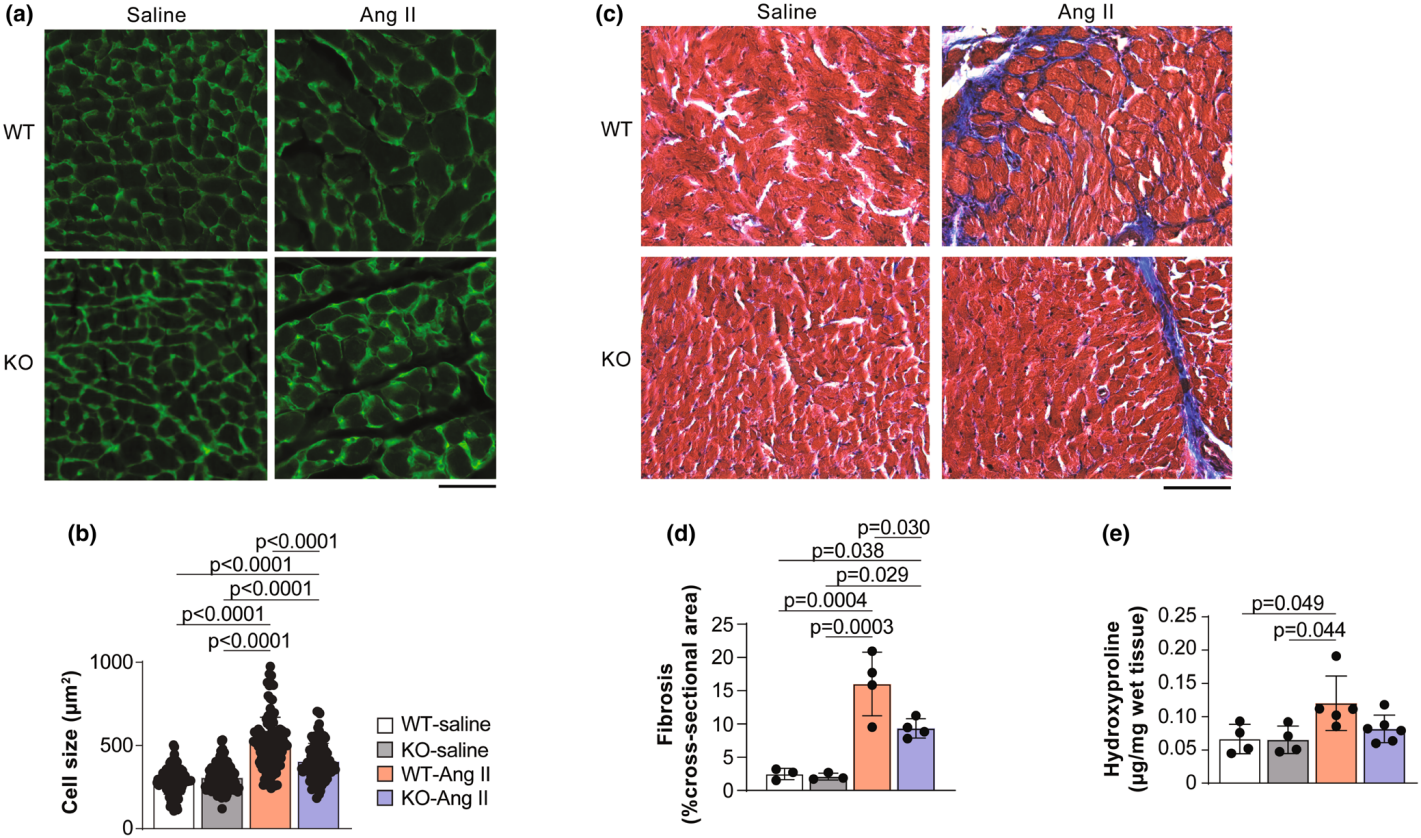
*Rbm20* KO attenuates Ang II‐induced cardiac remodeling. (a) Wheat germ agglutinin staining of heart sections for cardiomyocyte size measurement. Scale bar, 50 μm. (b) Quantification of cell size. *n* = 106–119 total cells from 3 animals per group. (c) Trichrome staining of heart sections. Scale bar, 100 μm. (d) Quantification of fibrosis area from trichrome staining. *n* = 3–4 animals per group. (e) Hydroxyproline assay to measure the collagen content in hearts. *n* = 4–6 animals per group. Bar graphs show mean ± SD. Data points represent measurements from individual animals. Significance determined by one‐way ANOVA followed by the Tukey post hoc test.

To further confirm reduced hypertrophy in Ang II‐treated *Rbm20* KO rats, cardiomyocyte size was determined by wheat germ agglutinin staining. Cardiomyocyte size was similar in both the WT and *Rbm20* KO groups without Ang II treatment (Figure 7A,B). While Ang II treatment led to cellular hypertrophy in both WT and *Rbm20* KO rats, the effect was significantly blunted in animals lacking RBM20 (Figure 7A,B). Trichrome staining revealed increased fibrotic areas in both treated WT and KO groups, and more fibrosis was observed in the Ang II‐treated WT group than in the Ang II‐treated *Rbm20* KO group (Figure 7C,D). Collagen content measured with the hydroxyproline assay further confirmed a significant increase in collagen in Ang II‐treated WT, but not in KO rats, hearts compared to that in their respective control groups (Figure 7E). These results suggest that the modulation of titin size in VSMCs reduces cardiac hypertrophy in response to Ang II treatment. Moreover, our data provide proof‐of‐concept evidence that targeting titin‐based VSMC tone through the RBM20‐titin axis may be beneficial for the treatment of cardiovascular diseases caused by or involving increased peripheral vascular resistance.

## 4 DISCUSSION

The novel findings of this study are that titin size regulates the stiffness of VSMCs and that reducing titin‐based VSMC stiffness ameliorates increased arterial stiffness in the context of elevated blood pressure. The increased titin size was observed in the aortic smooth muscle of *Rbm20* KO rats. Larger titin in smooth muscle reduced the stiffness of isolated VSMCs, as well as arterial stiffness in the context of Ang II‐induced hypertension but not at baseline. We found that *Rbm20* KO did not alter arterial ECM proteins and only minimally impacted Ang II‐associated changes in actomyosin cross‐bridges and vascular remodeling, further supporting a role for titin‐based VSMC stiffness in determining arterial stiffness under conditions of elevated blood pressure. Furthermore, we show that the reduced arterial stiffness in *Rbm20* KO rats lessens Ang II‐induced cardiac hypertrophy and fibrosis.

Remodeling and changes in the composition of the ECM are the most common mechanisms thought to underlie alterations in vascular stiffness. Increases in collagen and decreases in elastin have been reported in numerous studies of aging vessels that are responsible for increased arterial stiffness (Nilsson, 2014; Qiu et al., 2007; Sehgel, Vatner, & Meininger, 2015; Smulyan et al., 2016; Sun, 2015). However, several studies have demonstrated that a lack of increased collagen was observed in hypertensive vessels (Gorp et al., 2000; Sehgel, Sun, et al., 2015). In an aging monkey study, no changes in collagen content, but decreases in elastin were observed with elevated aortic stiffness (Qiu et al., 2007). Notably, studies of human essential hypertension demonstrated that the vascular collagen content was similar to or unchanged compared to age‐matched normotensive patients (Faber & Møller‐Hou, 1952; Hoshino et al., 1995). In this study, we also observed no change in the thickness of the adventitia layer in Ang II‐induced hypertensive *Rbm20* KO rats. While the Ang II‐induced increase in aortic wall thickness was blunted in *Rbm20* KO compared to WT rats, it seems likely that the increased compliance of VSMCs in KO rats plays a role in decreased arterial stiffness in these animals. There are two general assumptions that the contribution of VSMCs to vessel stiffness is associated with the degree of active tone in the cell, or independent of contractile tone, or the stiffening of the VSMCs themselves, which are controlled by the changes in the cellular cytoskeletal components of VSMCs. Numerous studies have suggested that intrinsic stiffness is an important functional property to both contractile and non‐contractile cell types (Barry et al., 2015; Cross et al., 2008; Trache et al., 2005; Wu et al., 2010). AFM has been applied to measure the stiffness of isolated individual VSMCs. A major increase in VSMC stiffness has been found in hypertensive and aging animal models along with the increased aortic stiffness (Qiu et al., 2010; Sehgel, Sun, et al., 2015; Zhu et al., 2012), which suggests that VSMC stiffness may be an indispensable contributor to the elevated vascular stiffness, and thus, trigger searches for a deeper understanding of mechanisms and factors that regulate the intrinsic stiffness of VSMCs. The increased VSMC stiffness was shown to be associated with the upregulated expression of cytoskeletal proteins in VSMCs (Qiu et al., 2010; Sehgel et al., 2013; Sehgel, Sun, et al., 2015; Sehgel, Vatner, & Meininger, 2015). On the other hand, it has also been observed that the stiffness of VSMCs is dynamic and oscillates with time (Sehgel et al., 2013), which implies that the dynamic properties of molecular motors or cytoskeletal polymerization and depolymerization may be involved in the regulation of VSMC stiffness. However, no such study has been done to search for the role of titin in the stiffness of VSMCs.

It has been shown in the work on rat cardiac muscle that titin is one of the major contributors to the passive stiffness of the myocardium, and variation in titin size‐based stiffness of the myocytes is likely to translate into variation in myocardial stiffness (Hamdani & Paulus, 2013). Here we suggest that titin size‐based stiffness may also regulate the stiffness of VSMCs. Titin has been identified in smooth muscles and potentially contributes to structural integrity by organizing thick filaments in the contractile apparatus and regulating the actin cytoskeleton in smooth muscle cells (Ayoob et al., 2000; Chi et al., 2005; Kim & Keller, 2002). Moreover, smooth muscle titin was suggested to provide smooth muscle tissue elasticity in the developing aorta where it is expressed at higher levels (Chi et al., 2005; Labeit et al., 2006). In smooth muscle, titin undergoes a developmental isoform transition. A full‐size titin transcript was detected in the human fetal aorta, while a truncated titin isoform including about 80 exons was detected in the human adult aorta (Labeit et al., 2006). RBM20 is known to regulate developmental titin isoform transition in the heart (Guo et al., 2012). The titin gene contains 364 exons. In the heart with RBM20 expression, cardiac titin undergoes extensive exon usage in the regions corresponding to the middle Ig and PEVK regions, from exon 50 to exon 219, to produce the major two classes of isoforms, N2BA and N2B (Bang et al., 2001; Cazorla et al., 2000; Guo et al., 2018; Stroik et al., 2024). The larger N2BA isoforms are mainly expressed in the fetal heart, and the smaller N2B isoform becomes predominant in the adult heart due to splicing inhibition of RBM20 (Maimaiti et al., 2021). Thus, we hypothesized that RBM20 regulates titin size in smooth muscle in a similar way as it does in cardiac muscle. Since smooth muscle titin reportedly undergoes a developmental isoform transition, from a full‐size isoform to a truncated smaller isoform (Labeit et al., 2006), it is possible that RBM20 controls the titin isoform transition in smooth muscle. Here, we observed the largest titin expression in the rat adult aorta, and the *Rbm20* KO aortic titin size is larger than the WT aortic titin, which indicates that RBM20 indeed plays a role in regulating smooth muscle titin splicing. Furthermore, by using different pairs of titin exon primers, we also confirmed increased exon inclusion in *Rbm20* KO smooth muscle. In primary cell cultures of VSMCs, the stiffness is significantly lower in *Rbm20* KO compared to WT VSMCs, and restoring RBM20 expression returned stiffness to levels comparable to those in WT VSMCs. Meanwhile, we also observed that in a hypertensive rat model induced by Ang II, the arterial stiffness was significantly reduced in *Rbm20* KO rats.

Lastly, Ang II‐induced hypertension is associated with cardiac remodeling. This was evident in Ang II‐treated WT rats as increases in LV mass and cardiomyocyte size. Notably, although Ang II treatment also promoted myocardial hypertrophy to some extent in *Rbm20* KO rats, it was not as severe as in WT animals. This finding suggests that increased titin‐based VSMC compliance in *Rbm20* KO rats can play a protective role in hypertension‐induced cardiac remodeling. Nevertheless, we were not able to dissociate the contributions of increased arterial and myocardial compliance to this outcome. Future studies will be necessary to establish the specific role that decreased titin‐based VSMC and arterial stiffness play in cardiac remodeling secondary to hypertension. Modulating RBM20 activity to regulate titin isoform expression is currently being explored to alleviate elevated myocardial stiffness in heart failure with preserved ejection fraction (HFpEF) (Methawasin et al., 2016; Radke et al., 2021). Our findings suggest that a similar strategy targeting the RBM20‐titin axis to fine‐tune VSMC stiffness could alleviate hemodynamic stress on the heart and lower the risk of end‐organ damage in the context of hypertension.

## AUTHOR CONTRIBUTIONS

WG conceived and designed the research. CZ and TB performed the experiments. WG, CZ, TB, and ZG analyzed the data, interpreted the results of the experiments, and prepared the figures. WG, CZ, and ZG drafted the manuscript, edited it, and revised it. WG, CZ, TB, and ZG approved the final version of the manuscript.

## FUNDING INFOMATION

This work was supported by the NIH NIGMS P20GM103432; HL148733; R03HD101870; the American Heart Association 16BGIA27790136, 19TPA34830072, and 23TPA1069731; USDA‐Hatch WIS04005.

## CONFLICT OF INTEREST STATEMENT

No conflicts of interest, financial, or otherwise, are declared by the authors.

## ETHICS STATEMENT

This study was conducted in accordance with the guidelines of the Institutional Animal Care and Use Committee of the University of Wyoming (Approval Number: 20180206BC00293).

## Supporting information


Table S1.

## Data Availability

Data will be made available upon reasonable request from the corresponding author.
