# Supplementary material for: Titin is a new factor regulating arterial stiffness through vascular smooth muscle cell tone in male rats
Source: Physiol Rep. 2025 Mar 22;13(6):e70270. doi: 10.14814/phy2.70270 (PMC11928681; doi:10.14814/phy2.70270)
Supplement: Supplementary file 1 — Table S1. [file PHY2-13-e70270-s001.docx]

**Titin is a new factor regulating arterial stiffness through vascular smooth muscle tone in male rats**

## Chaoqun Zhu^1^, Terrance Bishop^2^, Zachery R. Gregorich^3,4^, and Wei Guo^1,3,4*^

^1^Department of Animal Sciences, University of Wyoming, Laramie, WY 82071

^2^Colorado State University, Fort Collins, CO 80523

^3^Department of Animal and Dairy Sciences, University of Wisconsin-Madison, Madison, WI 53706

^4^Cardiovascular Research Center, University of Wisconsin-Madison, Madison, WI 53706

**Table S1. RT-PCR primers for the analysis of titin mRNA splicing.**

| Pair 1 (P1): Exon 120-122 | Forward: CACGTTGCAGTTTCCAAAAA |
| --- | --- |
|  | Reverse: TGGGAATAGGAACTGGCTTC |
| Pair 2 (P2): Exon 146-148 | Forward: CCTGTGCCTGAGAAGAAGGT |
|  | Reverse: AGCCACAGGAACTGGGACTA |
| Pair 3 (P3): Exon 148-150 | Forward: AGCCTAAGGTGCCAAAACCT |
|  | Reverse: GAGGAACCACCGCTTTCTTA |
| Pair 4 (P4): Exon 194-196 | Forward CCCGAAGACCAAGAAACTGT |
|  | Reverse: TTAGGTGGTGCCGCTGGT |
